# Obesity Impairs γδ T Cell Homeostasis and Antiviral Function in Humans

**DOI:** 10.1371/journal.pone.0120918

**Published:** 2015-03-18

**Authors:** Anne E. Costanzo, Kristen R. Taylor, Shelley Dutt, Peggy P. Han, Ken Fujioka, Julie M. Jameson

**Affiliations:** 1 California State University San Marcos, Department of Biology, 333 South Twin Oaks Drive, San Marcos, CA 92096, United States of America; 2 The Scripps Research Institute, Department of Immunology and Microbial Sciences, 10550 North Torrey Pines Rd, La Jolla, CA 92037, United States of America; 3 PHDataGroup 8189 Califa Court, San Diego, CA 92119, United States of America; 4 Scripps Clinic Research Center, 11025 N. Torrey Pines Rd., La Jolla, CA 92037, United States of America; University of Iowa, UNITED STATES

## Abstract

Obese patients are susceptible to increased morbidity and mortality associated with infectious diseases such as influenza A virus. γδ T cells and memory αβ T cells play key roles in reducing viral load by rapidly producing IFN-γ and lysing infected cells. In this article we analyze the impact of obesity on T lymphocyte antiviral immunity. Obese donors exhibit a reduction in γδ T cells in the peripheral blood. The severity of obesity negatively correlates with the number of γδ T cells. The remaining γδ T cells have a skewed maturation similar to that observed in aged populations. This skewed γδ T cell population exhibits a blunted antiviral IFN-γ response. Full γδ T cell function can be restored by potent stimulation with 1-Hydroxy-2-methyl-buten-4yl 4-diphosphate (HDMAPP), suggesting that γδ T cells retain the ability to produce IFN-γ. Additionally, γδ T cells from obese donors have reduced levels of IL-2Rα. IL-2 is able to restore γδ T cell antiviral cytokine production, which suggests that γδ T cells lack key T cell specific growth factor signals. These studies make the novel finding that the γδ T cell antiviral immune response to influenza is compromised by obesity. This has important implications for the development of therapeutic strategies to improve vaccination and antiviral responses in obese patients.

## Introduction

Obesity has reached epidemic proportions in the United States where greater than one third of adults are currently obese [1]. The clinical impact of obesity is substantial with adverse effects on health and life expectancy due to co-morbidities including type 2 diabetes, insulin resistance, and increased susceptibility to infection. In fact, obesity is an independent risk factor for increased hospitalization and death associated with respiratory viruses, such as the 2009 influenza A H1N1 pandemic [2–5]. Defects in primary and secondary αβ T cell responses to influenza and reduced function of epithelial γδ T cells have been identified in murine models of obesity [6–8]. Less is known about how obesity impacts influenza-specific T cell responses in humans including Vγ9Vδ2 T cells, which make up a sizeable proportion of the antiviral T cells able to rapidly respond to influenza virus [9–11].

Prior to the time required for conventional primary αβ T cells responses to develop, Vγ9Vδ2 T cells induce potent antiviral effector responses to influenza-infected cells [9–12]. They represent the predominant γδ T cell subset in human peripheral blood making up 1–10% of peripheral blood T lymphocytes. Vγ9Vδ2 T cells normally reside in the peripheral blood and lymphoid organs where they undergo maturation from naïve T cells to central memory T cells to effector memory T cells and finally T effector memory cells with CD45RA+ (TEMRA) [13]. Vγ9Vδ2 T cells play key roles in host defense via the production of IFN-γ and lysis of target cells infected with pathogens, including influenza A, Mycobacterium tuberculosis, HIV and EBV [11,14–16]. Unlike conventional αβ T cells that recognize peptide associated with MHC, human Vγ9Vδ2 T cells are activated by phosphorylated metabolites from microbes and stressed cells[17,18]. Although the antigen(s) involved in Vγ9Vδ2 T cell activation by influenza virus-infected cells is still unknown, it may be a virus-induced cellular phosphorylated metabolite. Our group and others have demonstrated that Vγ9Vδ2 T cells exhibit broad cross-reactive responses to cells infected with influenza viruses of all strains and subtypes known to infect humans [9], including the H1N1 pandemic strain [11]. Memory Vγ9Vδ2 T cells have been shown to migrate to the site of infection and perform effector functions that reduce disease severity and mortality in a humanized mouse model of influenza virus infection [10,12]. The cross-reactive and rapid nature of Vγ9Vδ2 T cell responses to influenza makes them an attractive target for therapy.

Obesity is associated with an increased susceptibility to both viral and bacterial pathogens, suggesting that immunity is compromised [7]. However, it is unknown how obesity impacts influenza-specific T cell responses in humans. Here we make the novel finding that Vγ9Vδ2 T cells are reduced in the peripheral blood of obese donors. We show that the remaining Vγ9Vδ2 T cells in obese donors exhibit enhanced differentiation to T effector memory populations and an aberrant effector response to influenza infection. Obesity does not fully suppress the ability of Vγ9Vδ2 T cells to function, as the potent phosphoantigen, 1-Hydroxy-2-methyl—buten-4yl 4-diphosphate (HDMAPP), is able to stimulate IFN-γ production by Vγ9Vδ2 T cells isolated from obese patients. Vγ9Vδ2 T cell dysfunction in obesity can be reversed with the addition of IL-2 signaling during influenza infection, suggesting that there may be a lack, or suppression, of appropriate cytokine reception in the obese environment. These findings represent novel therapeutic strategies to improve γδ T cell function in obese patients and lessen the severity of influenza infection.

## Research Design and Methods

### Human Subjects

Acquisition of blood samples and all scientific studies was reviewed and approved by the Institutional Review Board of Scripps Health (HSC-09-5116). Donors were enrolled at Scripps Clinic and the Scripps Research Institute Normal Blood Donor Services. Written informed consent was obtained from all donors. We restricted enrollment to subjects between the ages of 18 and 65 due to declining Vγ9Vδ2 T cell numbers in patients over 80 years of age [19,20]. Patients with a body mass index (BMI) of 20–25 were considered non-obese and subjects with a BMI equal to or greater than 30 were categorized as obese. Demographics for all subject samples are presented in Table 1.

**Table 1:** Demographics of donor samples.

	NOD	OD
**Age (yrs)**	**34 (22–52)**	**46 (19–67)**
**BMI (kg/m** ^**2**^)	**24**	**34**
**Females**	**13**	**9**
**Males**	**5**	**6**
**White**	**16**	**12**
**African American**	**0**	**0**
**Asian**	**2**	**0**
**Native American**	**0**	**0**
**Hispanic**	**0**	**0**
**Unknown**	**0**	**3**

Patients with a body mass index (BMI 20–25) were considered non-obese donors (NOD) and donors with a BMI equal to or greater than 30 were categorized as obese donors (OD).

### Cell Isolation and Staining

Whole blood was obtained from donors and peripheral blood mononuclear cells (PBMC) were isolated using Ficoll-Hypaque (VWR, Radnor, PA) density gradient centrifugation as previously described [9]. Isolated cells were stained with monoclonal antibodies immediately or post-culture with various stimuli. PBMC were stained with fluorescent-labeled antibodies CD3 (UCHT1), αβ TCR (IP26), Vδ2 (B6), CD4 (OKT4), CD8 (RPA-T8X2), CD69 (FN50), CD45RA (HI100), IFN-γ (B27), CD28, CD54 (HCD54), CCR5 (HEK/1/85a) (Biolegend, San Diego, CA), CD27 (LF.7F9) (eBioscience, San Diego, CA), CD25 (2A3) (BD Biosciences, San Diego, CA), NKG2D (1D11), and Granzyme B (GB11). Annexin-V and PI staining was performed using an Annexin-V Apoptosis Detection Kit (BD Biosciences).

### Stimulation of cells with HDMAPP

HDMAPP was purchased from Echelon (Salt Lake City, UT). 4.0x10^6^ PBMC in cRPMI 1640 containing 10% FCS were stimulated with 0.032μM or 0.316μM HDMAPP for 8 hours at 37°C. 5μg/ml brefeldin A (Sigma, St. Louis, MO) was added for the final 4 hours of stimulation. Following stimulation, cells were stained with antibodies using the BD Cytofix/Cytoperm Fixation/Permeabilization Kit (BD Biosciences), data was acquired on a LSR-II flow cytometer and data was analyzed by FlowJo software (Tree Star, Inc., Ashland, OR).

### Viruses and viral infection

Influenza A virus, A/Puerto Rico/8/34 (H1N1), was generously provided by Linda Sherman and propagated as previously described [21]. Virus was added to 4.0x10^6^ PBMC at a multiplicity of infection (MOI) of 5 for 8 hours at 37°C. 5μg/ml brefeldin A (Sigma, St. Louis, MO) was added for the final 4 hours of stimulation. Cells were stained with antibodies using the BD Cytofix/Cytoperm Kit, data acquired on a LSR-II flow cytometer and analyzed by FlowJo software (Tree Star, Inc., Ashland, OR). Alternatively, human monocytic U937 cells (ATCC, Manassas, VA) were infected with H1N1 influenza virus at a MOI of 5 for 19 hours at 37°C. Following incubation, U937 target cells were washed and incubated at an effector to target (E:T) ratio of 1:10 with 4.0x10^6^ PBMC effectors in RPMI containing 10% FCS for 8 hours at 37°C. 5μg/ml brefeldin A (Sigma, St. Louis, MO) was added for the final 4 hours of stimulation, cells were stained with antibodies using the BD Cytofix/Cytoperm Fixation/Permeabilization Kit (BD Biosciences), data acquired on a LSR-II flow cytometer and analyzed by FlowJo software (Tree Star, Inc., Ashland, OR). In some experiments 50U/ml IL-2 (NIH) was added to infected and uninfected cells.

### Statistics

All statistical analysis of Student’s unpaired *t* test and *f* test for variance were conducted using Microsoft Excel and Prism Graph Pad software. All findings are considered significant at P < 0.05.

## Results

### Vγ9Vδ2 T cells are reduced in obese subjects

**Fig 1:**
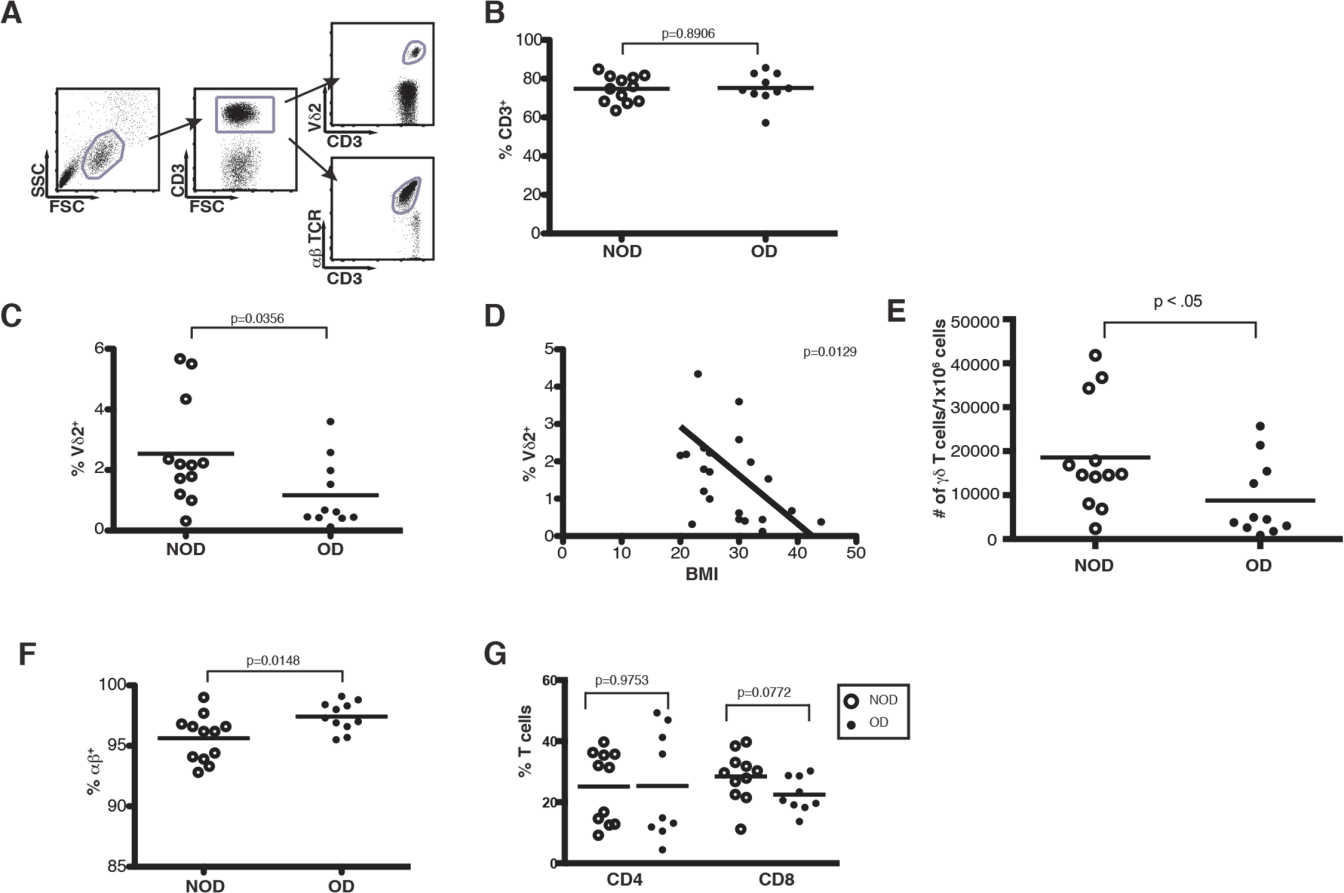
Obesity induces a dramatic loss of Vγ9Vδ2 T cells. PBMC isolated from whole blood of obese and non-obese donors were analyzed by flow cytometry. (A) Gating strategy of peripheral T cell subsets used to examine CD3^+^, αβ^+^, and Vδ2^+^ T cells in non-obese and obese donors. (B) Percentage of total T cells (CD3^+^ cells) in NOD and OD. Each dot represents an individual donor and horizontal lines represent the mean. (C) Percentage of Vδ2^+^ T cells in NOD and OD. Median 2.715% NOD and 0.618% OD. (D) Correlation of body mass index (BMI) to Vδ2^+^ T cell percentages. (E) Number of γδ T cells per 1x10^6^ human PBMC in NOD and OD. Mean 18,576 NOD and 8,783 OD. (F) Percentage of αβ ^+^ T cells in NOD and OD. Median 96.2% NOD and 97.3% in OD. (G) Percentage of CD4^+^ and CD8^+^ αβ T cells in NOD and OD. P-values calculated from Pearson’s unpaired Student’s *t*-test.

Due to the increased susceptibility of obese patients to more severe influenza virus infection, we investigated how obesity impacts known regulators of influenza infection such as T lymphocytes. PBMC were isolated from obese donors (OD) (BMI ≥ 30) and non-obese donors (NOD) (BMI ≤ 20–25) and analyzed for the composition of T lymphocyte populations in the blood (Table 1, Fig. 1). Cumulative data from obese and non-obese donors shown in Fig. 1 indicate that there are comparable percentages of total CD3^+^ T cells in the PBMC (Fig. 1). However, within the CD3^+^ T cell compartment obese donors demonstrate a skewed distribution of αβ versus γδ TCR-bearing cells. Vγ9Vδ2 T cells are reduced with a 4.5 fold reduction from 2.715% in non-obese donors to 0.618% in obese donors (Fig. 1). In fact, there is a significant inverse relationship between body mass index (BMI) and the percentage of Vγ9Vδ2 T cells in the PBMC (Fig. 1). The overall number of Vδ2+ cells in the PBMC of obese donors is also significantly reduced (Fig. 1). Conversely, the proportion of αβ T cells increases from 96.2% in non-obese donors to 97.3% in obese donors, but there is no significant change in CD4 or CD8 αβ T cell distribution (Fig. 1). Overall, obesity is associated with reduced Vγ9Vδ2 T cells, while αβ T cells are sustained and even increased.

### Reduced activation and increased differentiation of Vγ9Vδ2 T cells in obese donors

**Fig 2:**
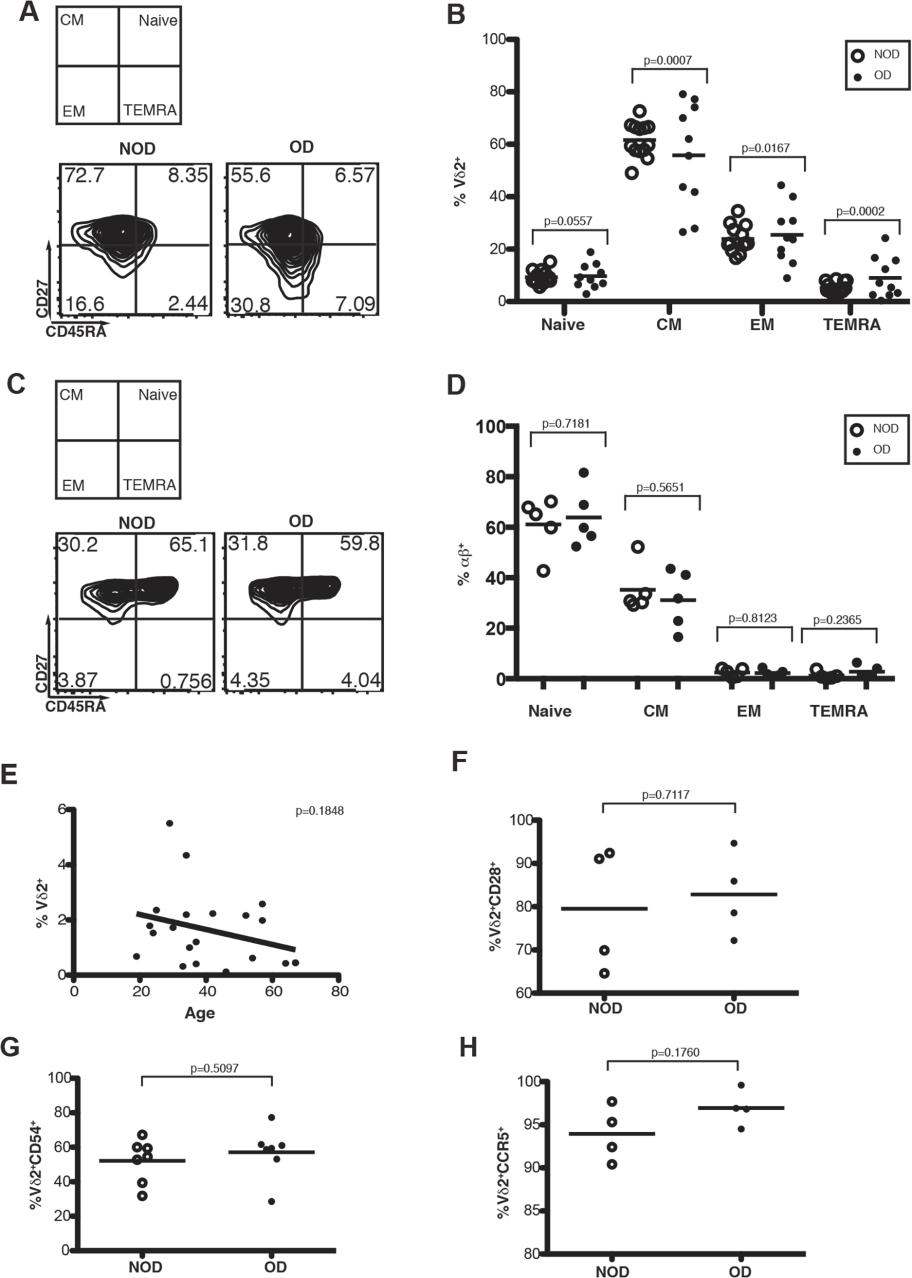
Obesity induces an aging Vγ9Vδ2 maturation phenotype. PBMC isolated from whole blood of obese and non-obese donors were analyzed by flow cytometry. (A) Representative plot of Vδ2^+^ naïve, central memory (CM), effector memory (EM) and T effector memory RA^+^ (TEMRA) subsets based on CD27 and CD45RA expression. Quadrants were determined using unstained negative controls. The number in each quadrant represents the percentage of Vδ2^+^ T cells. (B) Compilation of Vδ2^+^ T cell maturation in NOD and OD. P-values calculated from Pearson’s unpaired Student’s *t*-test, n = 10. (C) Representative plot of αβ naïve, central memory (CM), effector memory (EM) and T effector memory RA^+^ (TEMRA) subsets based on CD27 and CD45RA expression. Quadrants were determined using unstained negative controls. The number in each quadrant represents the percentage of αβ T cells. (D) Compilation of αβ T cell maturation in NOD and OD. P-values calculated from Pearson’s unpaired Student’s *t*-test, n = 5. (E) Correlation of age to Vδ2^+^ T cell percentages. (F-H) % of Vδ2^+^ T cells expressing receptors including CD28, CD54, and CCR5. P-values calculated from Pearson’s unpaired Student’s *t*-test, n = at least 4.

The majority of Vγ9Vδ2 T cells in the peripheral blood are central memory cells, with a strong capacity for proliferation but weaker cytokine producing abilities [22]. CD27 and CD45RA have been previously identified as maturation markers for Vγ9Vδ2 T cells. CD27 is expressed by immature lymphocytes and central memory cells (CM), but not on differentiated effector memory lymphocytes (EM) or T effector memory CD45RA^+^ (TEMRA) lymphocytes [13,23]. To determine whether obesity impacts γδ T cell maturation we compared CD27 and CD45RA expression on Vγ9Vδ2 T cells in obese and nonobese donors. Obese donors exhibited higher percentages of terminally differentiated TEMRA cells at the expense of central memory cells (Fig. 2). TEMRA cells are a reproducibly small population representing approximately 1–10%, of Vγ9Vδ2 T cells. However, TEMRA populations in obese donors make up as many as 20% of total Vγ9Vδ2 T cells. This data implies that the obese environment leads to enhanced Vγ9Vδ2 T cell differentiation, while αβ T cell expression of CD27 and CD45RA differentiation markers are not significantly impacted by obesity (Fig. 2). Thus obesity induces increased differentiation of Vγ9Vδ2 T cells from central memory to T effector memory and TEMRA populations. Increased differentiation indicates that the antiviral function of Vγ9Vδ2 T cells may be impaired.

Since reduced numbers of Vγ9Vδ2 T cells have been previously observed in elderly subjects between the ages of 80 and 100 [19,20], we confined enrollment of subjects to those aged 18–65 (Table 1). As observed in previous studies using similarly aged subjects [19], the correlation between Vγ9Vδ2 T cell percentages in the PBMC and age is not significant in subjects aged 18–65 (Fig. 2E). In addition, no impairment of CD28, ICAM-1 (CD54) or CCR5 expression was observed, suggesting obesity does not impair the expression of these key receptors involved in Vγ9Vδ2 T cell co-stimulation or homing (Fig. 2).

### Vγ9Vδ2 T cells from obese subjects exhibit blunted antiviral IFN-γ responses

**Fig 3:**
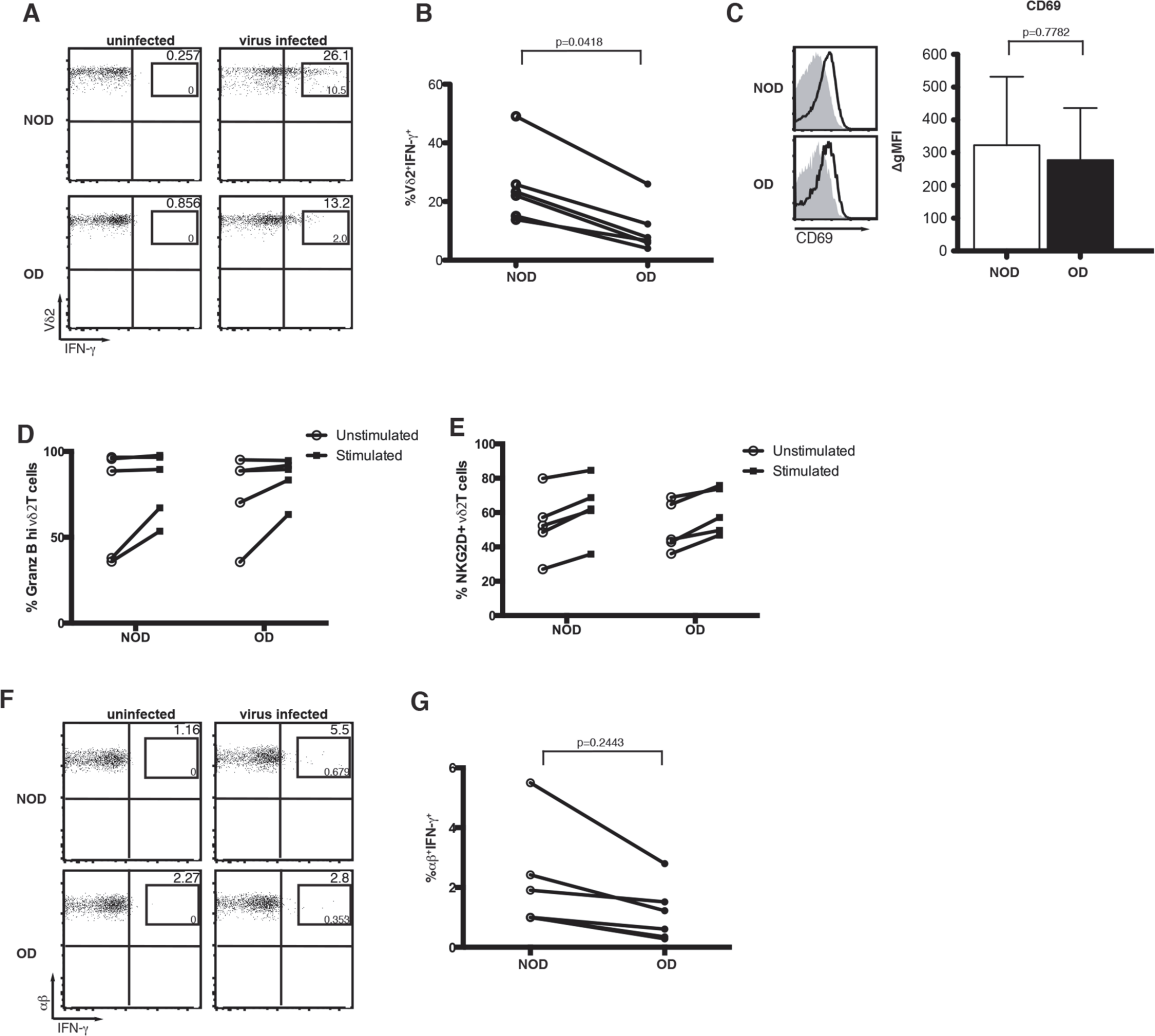
Vγ9Vδ2 T cell antiviral IFN-γ production is blunted in obese patients. PBMC were isolated from whole blood of OD and NOD, stimulated with influenza virus for 8 hours and analyzed for IFN-γ production and CD69 upregulation by flow cytometry. (A) Representative plot of IFN-γ^+^ production by Vδ2^+^ CD3^+^ gated cells. Large numbers in upper right quadrant represent the percent of Vδ2^+^ T cells producing IFN-γ, while small numbers inside the gate denote the percent of Vδ2^+^ T cells producing high amounts of IFN-γ. (B) Compilation of six separate experiments illustrating IFN-γ cytokine production by Vδ2 T cells. Lines connect paired donor sets for each experiment. (C) Vδ2^+^ T cells upregulate CD69 in NOD and OD following viral infection regardless of BMI. Histograms are gated on Vδ2^+^ CD3^+^ T cells. Grey histograms represent uninfected PBMC and black lines represent influenza-infected PBMC. Bar graph represents change in MFI +/- SD following influenza infection of PBMC from five OD and NOD. (D) Compilation of four separate experiments illustrating Granzyme B (D) or NKG2D (E) expression by unstimulated (open circles) or influenza virus stimulated (filled squares) Vδ2 T cells. Lines connect paired donor sets for each experiment. (F) Representative plot of IFN-γ^+^ production by αβ^+^ CD3^+^ gated cells. Large numbers in upper right quadrant represent the percent of αβ^+^ T cells producing IFN-γ, while small numbers inside the gate denote the percent of αβ^+^ T cells producing high amounts of IFN-γ. (G) Compilation of five separate experiments illustrating IFN-γ cytokine production by αβ T cells. Lines connect paired donor sets for each experiment. P-values calculated from Pearson’s unpaired Student’s *t*-test.

Work from our lab and others have demonstrated that Vγ9Vδ2 T cells rapidly respond to influenza infection by producing IFN-γ and lysing infected cells [9,11,12]. Obese patients are at an increased risk for more severe complications associated with influenza infection [2–4]. Thus, we examined whether the functional response of Vγ9Vδ2 T cells to influenza infection is impaired by obesity. Vγ9Vδ2 T cells from obese donors are significantly less responsive to H1N1 A/PR/8/34 (PR/8) influenza virus infection *in vitro* (Fig. 3). Both the number of cells producing IFN-γ and the amount of IFN-γ produced per cell are negatively impacted by obesity suggesting a blunting of the γδ T cell antiviral cytokine response. In fact, obese donors have very few Vγ9Vδ2 T cells able to produce high amounts of IFN-γ in response to influenza virus (Fig. 3). Upregulation of the activation marker CD69 is an early marker for T cell activation in response to influenza virus. Obesity does not impact the ability of Vγ9Vδ2 T cells to upregulate CD69, suggesting that early signaling is preserved in Vγ9Vδ2 T cells from obese subjects (Fig. 3). Additionally, Vγ9Vδ2 T cells have the ability to lyse influenza infected cells using a mechanism that depends greatly on NKG2D and granzyme B [11]. Vγ9Vδ2 T cells from obese donors exhibit similar expression levels of NKG2D and intracellular granzyme B as lean donors suggesting these key mechanisms of cytotoxicity are preserved (Fig. 3). NKG2D expression increased upon activation with influenza virus in both obese and lean donors showing that obese donors retain the ability to upregulate NKG2D in response to infection (Fig. 3). Thus the impairment in the remaining Vγ9Vδ2 T cells of obese donors is specific to antiviral cytokine secretion.

Influenza-specific αβ T cell responses could be detected after *in vitro* influenza infection. As expected, influenza-specific T cells make up a small proportion of the total αβ T cell population. In contrast the percentage of Vγ9Vδ2 T cells able to respond to influenza is much higher (Fig. 3). This underlines the point that although Vγ9Vδ2 T cells make up 1–2 percent of total T cells in the peripheral blood, the proportion that rapidly produces IFN-γ in response to influenza is comparable to that of memory αβ T cells. Obese donors exhibit a slight reduction in αβ T cell IFN-γ responses, however the change is not significant (Fig. 3).

### Vγ9Vδ2 T cells are less able to produce IFN-γ in response to nonobese APCs

**Fig 4:**
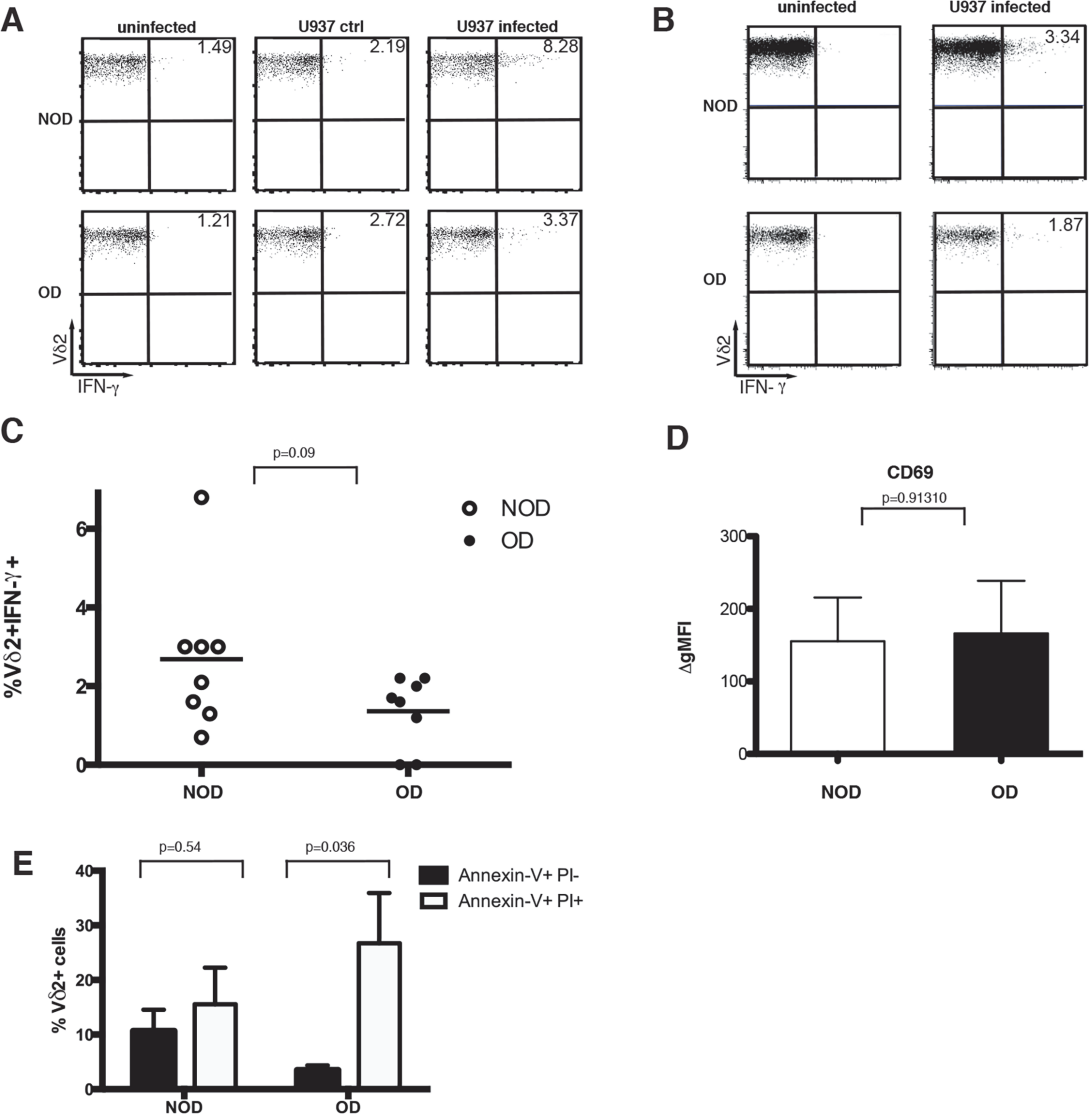
Vγ9Vδ2 T cells are more sensitive to apoptosis and have a reduced ability to produce IFN-γ in response to nonobese APCs. PBMC from non-obese (NOD) or obese (OD) donors were co-cultured with a monocytic cell line U937 to determine if the dysfunction was T cell intrinsic. (A, B) Representative plots of IFN-γ production by Vδ2^+^ CD3^+^ gated cells. PBMC were either cultured alone (uninfected), with U937 cells (U937 ctrl) or with influenza infected U937 cells (U937 infected). (C) Percentage of Vδ2^+^ T cells producing IFN-γ in NOD and OD in response to infected U937 cells (corrected for controls, n = 8). Horizontal lines represent the mean. (D) Change in MFI of CD69 expression on Vδ2^+^ CD3^+^ gated cells following 8 hour incubation with uninfected or infected U937 cells. Data represents five separate donor pairs and their standard deviation. P-values calculated from Pearson’s unpaired Student’s *t*-test. (E) Data represents the percentage of Vδ2^+^ T cells staining positive for Annexin-V but not PI (dark bars) or positive for both Annexin-V and PI (white bars) by flow cytometry (n = 5) with standard deviation.

Vγ9Vδ2 T cells in obese donors are able to upregulate NKG2D, CD69 and granzyme B suggesting that the influenza-infected antigen presenting cells (APCs) in the obese donor remain effective. To further investigate whether APC dysfunction in obesity contributes to the blunted Vγ9Vδ2 T cell responses to influenza virus, we utilized nonobese APCs to monitor activation and function of Vγ9Vδ2 T cells isolated from obese patients. In previous work [11], PR/8-infected U937 APCs activate Vγ9Vδ2 T cells to produce low levels of IFN-γ in a reproducible manner (Fig. 4). However, Vγ9Vδ2 T cells in obese donors have reproducibly diminished IFN-γ production even in the presence of virus-infected U937 APCs (overall mean = 2.7% lean, 1.4% obese) (Fig. 4). When the individual experiments are analyzed together the difference is just short of significance (p = 0.09). However, in each individual experiment the number of IFN-γ-producing γδ T cells in obese donors was reduced by half. Again CD69 upregulation is similar between obese and non-obese donors (Fig. 4). These results suggest that despite the presence of nonobese APCs, Vγ9Vδ2 T cell IFN-γ responses could not be fully restored.

To assess whether the Vγ9Vδ2 T cells themselves become apoptotic leading to their ultimate reduction in number, we used Annexin-V/PI staining to identify apoptotic Vγ9Vδ2 T cells from obese and lean donors. We observed a shift from Annexin-V+ PI- Vγ9Vδ2 T cells, toward Annexin-V+ PI+ Vγ9Vδ2 T cells in obese donors. Lean donors had a similar percent of their Vγ9Vδ2 T cells entering (Annexin-V+ PI-) and undergoing apoptosis (Annexin-V+ PI+), while obese donors exhibited a significant difference between their low numbers of Vγ9Vδ2 T cells entering apoptosis (Annexin-V+ PI-) as compared to their fully apoptotic cells (Annexin-V+ PI+) (P = 0.036). This data suggests that the Vγ9Vδ2 T cells in obese donors are more sensitive to cell death perhaps rendering them less able to produce cytokines.

**Fig 5:**
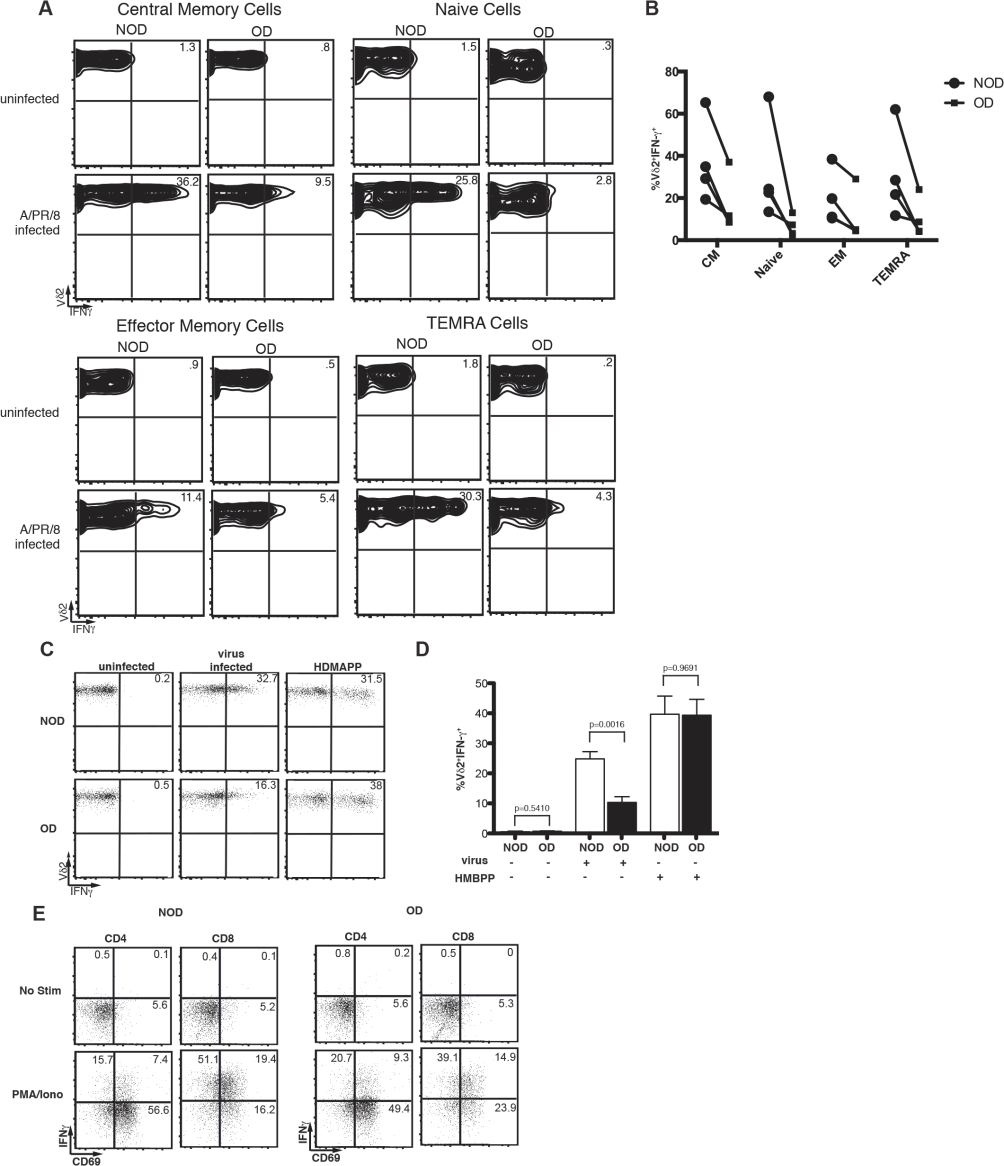
Strong ligation with HDMAPP induces IFN-γ production by Vγ9Vδ2 T cells from obese donors. Influenza virus was added to PBMC from non-obese (NOD) and obese (OD) subjects to induce IFN-γ production by Vδ2^+^ T cells and assess the level of IFN-γ production by various memory subsets. (A) Representative plots showing IFN-γ production by Vδ2^+^ cells from the PBMC of NOD and OD without infection or following virus infection. Cells were analyzed as naïve, central memory (CM), effector memory (EM) and T effector memory RA^+^ (TEMRA) subsets based on CD27 and CD45RA expression (B) Compilation of four separate experiments illustrating IFN-γ cytokine production by Vδ2 T cells. Lines connect paired donor sets for each experiment. Circles represent NOD and squares represent OD. (C-D) The Vγ9Vδ2 T cell ligand HDMAPP was added to PBMC from non-obese (NOD) and obese (OD) subjects to induce IFN-γ production by Vδ2^+^ T cells (C) Representative plots showing IFN-γ production by Vδ2^+^ cells from the PBMC of NOD and OD without infection, following virus infection, or upon activation with HDMAPP. (D) Compilation of HDMAPP stimulation in five separate donor sets with standard deviation. Mean percent of Vδ2^+^ CD3^+^ gated cells producing IFN-γ from NOD and OD. (E) Representative plots of PBMC from NOD and OD stimulated with or without PMA and ionomycin. Experiments are representative of at least three donor sets. P-values calculated from Pearson’s unpaired Student’s *t*-test.

Together the increase in Vγ9Vδ2 TEMRA cells within obese patients and the increased sensitivity of Vγ9Vδ2 T cells to apoptosis suggest a terminal differentiation that results in Vγ9Vδ2 T cell dysfunction and death. To identify whether TEMRA cells are the only Vγ9Vδ2 T cells that lose the ability to produce large amounts of IFN-γ, we examined the ability of naïve, central memory, EM, and TEMRA cells to produce IFN-γ in response to influenza infection. Obesity results in a reproducible reduction in IFN-γ production during all of the states of T cell maturation with naïve T cells showing significance when the experiments are analyzed together (p = 0.03) (Fig. 5).

### Obesity does not impair Vγ9Vδ2 T cell IFN-γ production in response to strong ligands

Vγ9Vδ2 T cells recognize and are activated by non-prenylpeptidic antigens such as those in the mevalonate pathway [18]. One potent activator is HDMAPP, produced by many types of bacteria [24], although it is still somewhat controversial how HDMAPP activates Vγ9Vδ2 T cells. Activation by other phosphoantigens such as isopentenyl pyrophosphate (IPP) requires cell-cell contact, but does not require cellular processing [25,26]. To determine if a strong stimulus, such as that delivered by the phosphoantigen HDMAPP, can induce normal IFN-γ production by Vγ9Vδ2 T cells isolated from obese subjects, PBMC were stimulated *in vitro* with HDMAPP and IFN-γ production examined. Strikingly, HDMAPP fully restored IFN-γ responses in obese donors at all concentrations of HDMAPP tested (Fig. 5 and data not shown). Not only were the number of IFN-γ-producing Vγ9Vδ2 T cells the same in obese donors as compared to their lean counterparts, but also the ability to produce high levels of IFN-γ was similar between obese and lean donors. As expected IFN-γ production and CD69 upregulation by αβ T cells in response to PMA and ionomycin were comparable between obese and lean donors (Fig. 5). The restoration of Vγ9Vδ2 T cell function suggests that Vγ9Vδ2 TCR signaling remains functional in obesity. However, cytokine signals, such as signal 3, may be limiting or dysfunctional.

### IL-2 reverses dysfunctional Vγ9Vδ2 T cell responses in obese and non-obese subjects

**Fig 6:**
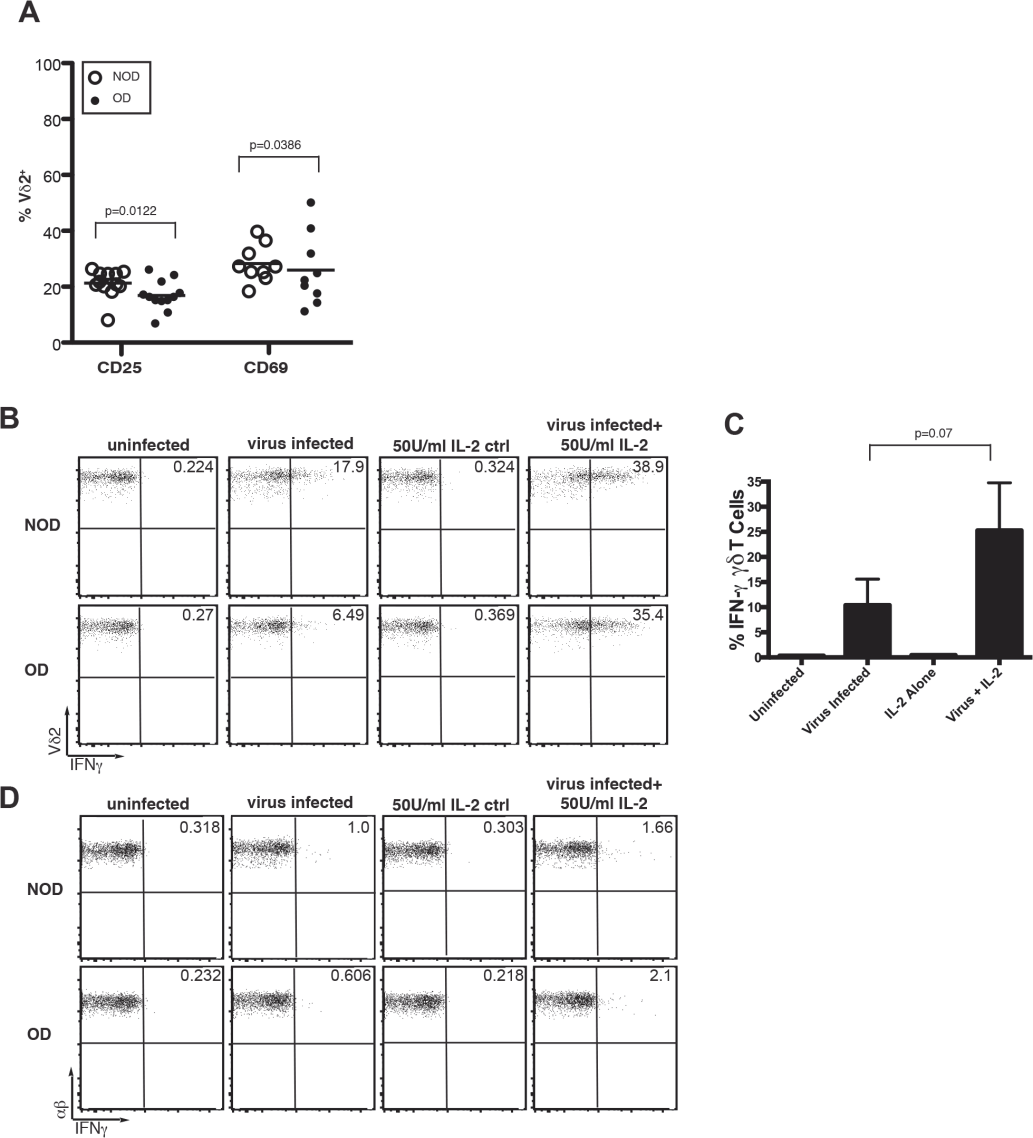
Addition of IL-2 restores effector function in Vδ2 T cells from obese donors. (A) Percentage of peripheral Vδ2^+^ T cells expressing CD25 and CD69. Each dot represents an individual non-obese (NOD) or obese (OD) donor and horizontal lines represent the mean. (B-D) IL-2 was added to the PBMC from NOD and OD to examine IFN-γ production by (B) Vδ2 T cells or (D) αβ T cells following influenza infection in the presence or absence of IL-2. Cells were infected for 8 hours prior to examination. (C) Compilation of IL-2 stimulation of OD PBMC in three separate experiments with standard deviation. Experiments are representative of three donor sets. P-values calculated from Pearson’s unpaired Student’s *t*-test.

IL-2 regulates Vγ9Vδ2 T cell proliferation and survival and enhances cytokine responses including IFN-γ production [18] [27]. Studies have identified reduced IL-2 levels in the serum of obese subjects [28]. We investigated whether obesity impacts the ability of T cells to express the IL-2 receptor alpha, CD25, homeostatically. In obese donors, CD25 and CD69 expression was slightly reduced in Vγ9Vδ2 T cells (Fig. 6), although no significant difference in expression was observed in αβ T cells (data not shown). Thus, obesity causes reduced expression of CD25 on Vγ9Vδ2 T cells, suggesting altered IL-2 cytokine reception in Vγ9Vδ2 T cells in obesity. With IL-2 levels limiting in obesity, we investigated whether Vγ9Vδ2 T cell antiviral functional responses could be restored by the addition of this cytokine. When IL-2 was added to the PBMC of obese donors during stimulation with influenza virus, we found that IL-2 successfully restored the ability of Vγ9Vδ2 T cells to produce IFN-γ (Fig. 6). The average fold-increase in IFN-γ producing cells after the addition of IL-2 was 2.5 fold over the addition of virus alone. When the experiments were grouped together this increase was just short of obtaining significance (P = 0.07), but increased IFN-γ production was observed in each individual experiment in a reproducible manner. IL-2 also improved Vγ9Vδ2 T cell IFN-γ production in response to influenza virus-infected U937 APCs (data not shown). αβ T cell IFN-γ production specific for influenza was slightly improved in both NOD and OD donors (Fig. 6). Comparison of the fold increase in Vδ2 T cell IFN-γ production in IL-2 treated PBMC in NOD and OD suggests Vδ2 T cells in obese patients are responsive to the addition of growth factor. These findings indicate that IL-2-promotes the activation of IFN-γ production by activated T cells in obese patients and that this pathway may be targeted to overcome γδ T cell dysfunction in obesity.

## Discussion

This study demonstrates the dramatic impact of obesity on a key T cell population in the peripheral blood. We demonstrate that Vγ9Vδ2 T cells become significantly reduced with increased BMI. Vγ9Vδ2 T cell numbers are a relatively minor population at birth, but become the predominant γδ T cell subset in the peripheral blood and secondary lymphoid organs around 7 years of age through peripheral antigen-driven expansion [29]. In adults the number of Vγ9Vδ2 T cells remains stable until 80–100 years of age when Vγ9Vδ2 T cells decline [19,20,29]. The data herein identify a premature reduction in Vγ9Vδ2 T cells in obese subjects prior to age 80. This has negative implications for antiviral immunity in obese subjects as γδ T cells have demonstrated roles in the immune response to infectious agents via cytokine production and cytolysis [9,11,14–16]. Vγ9Vδ2 T cells likely home from the peripheral blood to the lung upon influenza infection as has been shown in a humanized mouse model [10]. This suggests that reduced number and function of γδ T cells in obesity would exacerbate morbidity and mortality caused by influenza virus infection.

Beyond the loss in Vγ9Vδ2 T cell number, obesity accelerates γδ T cell differentiation. Obesity leads to an accumulation of more terminally differentiated memory γδ T cells. It is important to note that patients aged 80–100 years of age exhibit reduced expression of the maturation marker CD27 on Vγ9Vδ2 T cells [20]. Thus, obese patients experience premature differentiation of the Vγ9Vδ2 T cell compartment causing an increased prevalence of TEMRA cells similar to that observed in centennial subjects. TEMRA γδ T cells are known to home to sites of inflammation where they produce inflammatory cytokines such as IFN-γ [13]. In our studies the TEMRA γδ T cells are also limited in their ability to produce IFN-γ. The association of chronic inflammation in obesity with Vγ9Vδ2 T cell terminal differentiation and loss of central memory will be an important avenue for investigation. Recent studies have also correlated aging with a low level of chronic inflammation, providing further support to the hypothesis that obesity causes premature aging of Vγ9Vδ2 T cells [30,31]. It is interesting to speculate whether a prematurely aged Vγ9Vδ2 T cell compartment in an obese patient can be reversed upon weight loss or treatment with anti-inflammatory therapeutics.

Obesity induces metabolic exhaustion of epidermal γδ T cells in mice, resulting in defects in the ability of these cells to produce cytokines and growth factors upon activation [32]. The exhaustion is reversible upon removal of epidermal γδ T cells from the obese environment or treatment with anti-TNF-α antibodies [32]. Here our data show that obese subjects exhibit blunted production of IFN-γ by Vγ9Vδ2 T cells responding to influenza-infected cells. Potent TCR stimulation with a strong pyrophosphate antigen can countermand the loss of Vγ9Vδ2 T cell effector function within obese patients. Thus, it appears that the ability of Vγ9Vδ2 T cells to function remains intact in obesity; however strong stimulation is needed to circumvent T cell intrinsic defects in obese donors.

Taken together, reduced Vγ9Vδ2 T cell numbers, reduced IL-2Ra (CD25) expression, sensitivity to apoptosis, and accelerated terminal differentiation suggest that γδ T cells are lacking signals required for homeostatic proliferation and enhancement of cellular function. Obese subjects have reduced IL-2 levels in the serum [28] suggesting that IL-2 signals appropriate for the maintenance of Vγ9Vδ2 T cells are absent. Vγ9Vδ2 T cells are very responsive to IL-2 for metabolic activity that results in proliferation, increased cytokine production, and survival [18,33,34]. Additionally, IL-2 is able to restore normal proliferation to Vγ9Vδ2 cells in the absence of CD28 costimulation [35]. The ability of IL-2 to restore antiviral function of Vγ9Vδ2 T cells isolated from obese patients suggests that signal 3 is sufficient to improve IFN-γ transcription. IL-2 deficient mice have reduced levels of αβ T cells producing IFN-γ in response to virus infection further suggesting a key role for IL-2 in IFN-γ production [36]. Future studies will be important to investigate how IL-2 levels become reduced in obese patients. The sources of IL-2 in the peripheral blood include CD4 T cells, CD8 T cells, dendritic cells, NK cells or NK T cells so it will be important to delineate whether one or more of these cell types are suppressed [37]. It is possible that the IL-2 transcriptional repressor BLIMP1 is increased in T lymphocytes from obese patients, which could result in reduced IL-2 production. Our studies suggest that investigation into pyrophosphate antigens or cytokine-based therapeutic strategies may prove fruitful for improving antiviral responses in obese patients through the enhancement of Vγ9Vδ2 T cell TCR or cytokine signaling. With the increasing number of obese patients in the United States and abroad, changes that occur within the immune system need to be explored and novel strategies for improving immune function in obese patients developed.

## References

[1] National Center for Health Statistics (2008) Untitled-p4286: 1–574.

[2] Fezeu L, Julia C, Henegar A, Bitu J, Hu FB, Grobbee DE, et al (2011) Obesity is associated with higher risk of intensive care unit admission and death in influenza A (H1N1) patients: a systematic review and meta-analysis. Obesity Reviews 12: 653–659. Available: http://eutils.ncbi.nlm.nih.gov/entrez/eutils/elink.fcgi?dbfrom=pubmed&id=21457180&retmode=ref&cmd=prlinks. 10.1111/j.1467-789X.2011.00864.x 21457180

[3] Kwong JC, Campitelli MA, Rosella LC (2011) Obesity and respiratory hospitalizations during influenza seasons in Ontario, Canada: a cohort study. Clin Infect Dis 53: 413–421. 10.1093/cid/cir442 21844024 PMC3156143

[4] Nguyen-Van-Tam JS, Openshaw PJM, Hashim A, Gadd EM, Lim WS, Semple MG, et al (2010) Risk factors for hospitalisation and poor outcome with pandemic A/H1N1 influenza: United Kingdom first wave (May-September 2009). Thorax 65: 645–651. 10.1136/thx.2010.135210 20627925 PMC2921287

[5] Huttunen R, nen JSA (2012) Obesity and the risk and outcome of infection. Int J Obes Relat Metab Disord 37: 333–340. 10.1038/ijo.2012.62 22546772

[6] Smith AG, Sheridan PA, Tseng RJ, Sheridan JF, Beck MA (2009) Selective impairment in dendritic cell function and altered antigen-specific CD8 +T-cell responses in diet-induced obese mice infected with influenza virus. Immunology 126: 268–279. 10.1111/j.1365-2567.2008.02895.x 18754811 PMC2632688

[7] Karlsson EA, Beck MA (2010) The burden of obesity on infectious disease. Experimental Biology and Medicine 235: 1412–1424. 10.1258/ebm.2010.010227 21127339

[8] Taylor KR, Mills RE, Costanzo AE, Jameson JM (2010) γδ T Cells Are Reduced and Rendered Unresponsive by Hyperglycemia and Chronic TNFα in Mouse Models of Obesity and Metabolic Disease. PLoS ONE 5: e11422 10.1371/journal.pone.0011422.s005 20625397 PMC2896399

[9] Jameson JM, Cruz J, Costanzo A, Terajima M, Ennis FA (2010) A role for the mevalonate pathway in the induction of subtype cross-reactive immunity to influenza A virus by human gammadelta T lymphocytes. Cell Immunol 264: 71–77. 10.1016/j.cellimm.2010.04.013 20483407 PMC2905741

[10] Tu W, Zheng J, Liu Y, Sia SF, Liu M, Qin G, et al (2011) The aminobisphosphonate pamidronate controls influenza pathogenesis by expanding a γδ T cell population in humanized mice. Journal of Experimental Medicine 208: 1511–1522. 10.1086/500954 21708931 PMC3135369

[11] Qin G, Mao H, Zheng J, Sia SF, Liu Y, Chan P., et al (2009) Phosphoantigen‐Expanded Human γδ T Cells Display Potent Cytotoxicity against Monocyte‐Derived Macrophages Infected with Human and Avian Influenza Viruses. J INFECT DIS 200: 858–865. 10.1086/605413 19656068 PMC7110194

[12] Qin G, Liu Y, Zheng J, Ng IHY, Xiang Z, Lam KT, et al (2011) Type 1 Responses of Human Vγ9Vδ2 T Cells to Influenza A Viruses. J Virol 85: 10109–10116. 10.1128/JVI.05341-11 21752902 PMC3196408

[13] Dieli F, Poccia F, Lipp M, Sireci G, Caccamo N, Di Santo C, et al (2003) Differentiation of effector/memory Vδ2 T cells and migratory routes in lymph nodes or inflammatory sites. J Exp Med 198: 391–397. 10.1084/jem.20030235 12900516 PMC2194087

[14] Carding SR, Allan W, Kyes S, Hayday A, Bottomly K, Doherty PC (1990) Late dominance of the inflammatory process in murine influenza by gamma/delta + T cells. J Exp Med 172: 1225–1231. 2145388 10.1084/jem.172.4.1225 PMC2188600

[15] Boullier S, Dadaglio G, Lafeuillade A, Debord T, Gougeon ML (1997) Vδ1 T cells expanded in the blood throughout HIV infection display a cytotoxic activity and are primed for TNF-alpha and IFN-gamma production but are not selected in lymph nodes. J Immunol 159: 3629–3637. 9317163

[16] Häcker G, Kromer S, Falk M, Heeg K, Wagner H, Pfeffer K (1992) Vδ1+ subset of human gamma delta T cells responds to ligands expressed by EBV-infected Burkitt lymphoma cells and transformed B lymphocytes. J Immunol 149: 3984–3989. 1334108

[17] Kabelitz D (2011) γδ T-cells: cross-talk between innate and adaptive immunity. Cell Mol Life Sci 68: 2331–2333. 10.1007/s00018-011-0696-4 21541699 PMC11115158

[18] Morita CT, Jin C, Sarikonda G, Wang H (2007) Nonpeptide antigens, presentation mechanisms, and immunological memory of human Vγ9Vδ2 T cells: discriminating friend from foe through the recognition of prenyl pyrophosphate antigens. Immunol Rev 215: 59–76. 10.1111/j.1600-065X.2006.00479.x 17291279

[19] Argentati K, Re F, Donnini A, Tucci MG, Franceschi C, Bartozzi B, et al (2002) Numerical and functional alterations of circulating gammadelta T lymphocytes in aged people and centenarians. Journal of Leukocyte Biology 72: 65–71. 12101264

[20] Re F, Poccia F, Donnini A, Bartozzi B, Bernardini G, Provinciali M (2005) Skewed representation of functionally distinct populations of Vγ9Vδ2 T lymphocytes in aging. Experimental Gerontology 40: 59–66. 10.1016/j.exger.2004.09.008 15664733

[21] Wei C-H, Trenney R, Sanchez-Alavez M, Marquardt K, Woodland DL, Henriksen SJ, et al (2005) Tissue-resident memory CD8+ T cells can be deleted by soluble, but not cross-presented antigen. J Immunol 175: 6615–6623. 16272316 10.4049/jimmunol.175.10.6615

[22] Caccamo N, Meraviglia S, Ferlazzo V, Angelini D, Borsellino G, Poccia F, et al (2005) Differential requirements for antigen or homeostatic cytokines for proliferation and differentiation of human Vγ9Vδ2 naive, memory and effector T cell subsets. Eur J Immunol 35: 1764–1772. 10.1002/eji.200525983 15915537

[23] Gioia C, Agrati C, Casetti R, Cairo C, Borsellino G, Battistini L, et al (2002) Lack of CD27-CD45RA- Vγ9Vδ2+ T cell effectors in immunocompromised hosts and during active pulmonary tuberculosis. J Immunol 168: 1484–1489. 11801693 10.4049/jimmunol.168.3.1484

[24] Altincicek B, Moll J, Campos N, Foerster G, Beck E, Hoeffler J, et al (2001) Cutting edge: human gamma delta T cells are activated by intermediates of the 2-C-methyl-D-erythritol 4-phosphate pathway of isoprenoid biosynthesis. J Immunol 166: 3655–3658. 11238603 10.4049/jimmunol.166.6.3655

[25] Morita CT, Beckman EM, Bukowski JF, Tanaka Y, Band H, Bloom BR, et al (1995) Direct presentation of nonpeptide prenyl pyrophosphate antigens to human gamma delta T cells. Immunity 3: 495–507. 7584140 10.1016/1074-7613(95)90178-7

[26] Lang F, Peyrat MA, Constant P, Davodeau F, David-Ameline J, Poquet Y, et al (1995) Early activation of human Vγ9Vδ2 T cell broad cytotoxicity and TNF production by nonpeptidic mycobacterial ligands. J Immunol 154: 5986–5994. 7751641

[27] Kasahara T, Hooks JJ, Dougherty SF, Oppenheim JJ (1983) Interleukin 2-mediated immune interferon (IFN-gamma) production by human T cells and T cell subsets. J Immunol 130: 1784–1789. 6403613

[28] Aygun AD, Gungor S, Ustundag B, Gurgoze MK, Sen Y (2005) Proinflammatory Cytokines and Leptin Are Increased in Serum of Prepubertal Obese Children. Mediators of Inflammation 2005: 180–183. 10.1155/MI.2005.180 16106106 PMC1526468

[29] Parker CM, Groh V, Band H, Porcelli SA, Morita C, Fabbi M, et al (1990) Evidence for extrathymic changes in the T cell receptor gamma/delta repertoire. J Exp Med 171: 1597–1612. 2185330 10.1084/jem.171.5.1597 PMC2187908

[30] Hearps AC, Martin GE, Angelovich TA, Cheng W-J, Maisa A, Landay AL, et al (2012) Aging is associated with chronic innate immune activation and dysregulation of monocyte phenotype and function. Aging Cell 11: 867–875. 10.1111/j.1474-9726.2012.00851.x 22708967

[31] Cavanagh MM, Weyand CM, Goronzy JJ (2012) Chronic inflammation and aging: DNA damage tips the balance. Curr Opin Immunol 24: 488–493. 10.1016/j.coi.2012.04.003 22565047 PMC3423478

[32] Taylor KR, Mills RE, Costanzo AE, Jameson JM (2010) Gammadelta T cells are reduced and rendered unresponsive by hyperglycemia and chronic TNFalpha in mouse models of obesity and metabolic disease. PLoS ONE 5: e11422 10.1371/journal.pone.0011422 20625397 PMC2896399

[33] Hayday AC (2000) γδ cells: a right time and a right place for a conserved third way of protection. Annu Rev Immunol 18: 975–1026. 10.1146/annurev.immunol.18.1.975 10837080

[34] Jameson J, Havran WL (2007) Skin γδ T-cell functions in homeostasis and wound healing. Immunol Rev 215: 114–122. 10.1111/j.1600-065X.2006.00483.x 17291283

[35] Ribot JC, deBarros A, Mancio-Silva L, Pamplona A, Silva-Santos B (2012) B7-CD28 costimulatory signals control the survival and proliferation of murine and human γδ T cells via IL-2 production. The Journal of Immunology 189: 1202–1208. 10.4049/jimmunol.1200268 22732586

[36] Su HC, Cousens LP, Fast LD, Slifka MK, Bungiro RD, Ahmed R, et al (1998) CD4+ and CD8+ T cell interactions in IFN-gamma and IL-4 responses to viral infections: requirements for IL-2. J Immunol 160: 5007–5017. 9590250

[37] Boyman O, Sprent J (2012) The role of interleukin-2 during homeostasis and activation of the immune system. Nat Rev Immunol. 10.1038/nri3156 22343569

